# Cerebral organoids as tools to identify the developmental roots of autism

**DOI:** 10.1186/s13229-020-00360-3

**Published:** 2020-07-13

**Authors:** Wai Kit Chan, Rosie Griffiths, David J. Price, John O. Mason

**Affiliations:** Centre for Discovery Brain Sciences and Simons Initiative for the Developing Brain, University of Edinburgh, George Square, Edinburgh, EH8 9XD UK

**Keywords:** Autism spectrum disorder, Cerebral organoids, Embryonic brain development

## Abstract

Some autism spectrum disorders (ASD) likely arise as a result of abnormalities during early embryonic development of the brain. Studying human embryonic brain development directly is challenging, mainly due to ethical and practical constraints. However, the recent development of cerebral organoids provides a powerful tool for studying both normal human embryonic brain development and, potentially, the origins of neurodevelopmental disorders including ASD. Substantial evidence now indicates that cerebral organoids can mimic normal embryonic brain development and neural cells found in organoids closely resemble their in vivo counterparts. However, with prolonged culture, significant differences begin to arise. We suggest that cerebral organoids, in their current form, are most suitable to model earlier neurodevelopmental events and processes such as neurogenesis and cortical lamination. Processes implicated in ASDs which occur at later stages of development, such as synaptogenesis and neural circuit formation, may also be modeled using organoids. The accuracy of such models will benefit from continuous improvements to protocols for organoid differentiation.

## Introduction

Autism spectrum disorders (ASDs) are a group of neurodevelopmental disorders that affect as many as 1 in 59 children [17]. They are characterized by impairments in social interaction and communication and repetitive and restricted patterns of behavior, interests, or activities. While these symptoms can be found in any individual across the spectrum, the severity of the symptoms presented varies, ranging from very mild to very severe. Individuals with ASD may also present distinct combinations of comorbid features and diagnoses that are not part of the disorder they were diagnosed with, such as gastrointestinal symptoms, epilepsy, sleep disruptions, or motor disturbances. This clinical heterogeneity makes it difficult to find a unifying biological hypothesis to address all the features of ASD and the underlying genetic causes of ASDs are still under debate.

Studies of large family cohorts have identified at least 65 ASD risk genes with high confidence [46, 158] and hundreds more candidate genes. However, this only accounts for about 30% of ASD cases, the remainder having nonsyndromic idiopathic ASD in which the cause is unknown [44]. It is estimated that as many as 300-1000 genes could be targets for rare mutations which greatly increase the risk of ASD, potentially explaining some idiopathic cases [60, 125]. This extreme genetic heterogeneity makes it very difficult to map the relationship between genotype and phenotype in ASD [34]. However, recent work using network approaches suggests that autism risk genes converge on a small number of biological pathways and processes [33, 53, 97, 151]. Gene set enrichment analyses have shown that genes associated with ASD converge on pathways and processes that contribute to embryonic brain development, including chromatin remodeling, neurogenesis, and cortical lamination; neuronal physiological maintenance; and synaptic processes. The convergence of many ASD-risk genes on common molecular pathways may help explain how a genetically heterogeneous population of individuals exhibit similar symptoms.

**Fig. 1:**
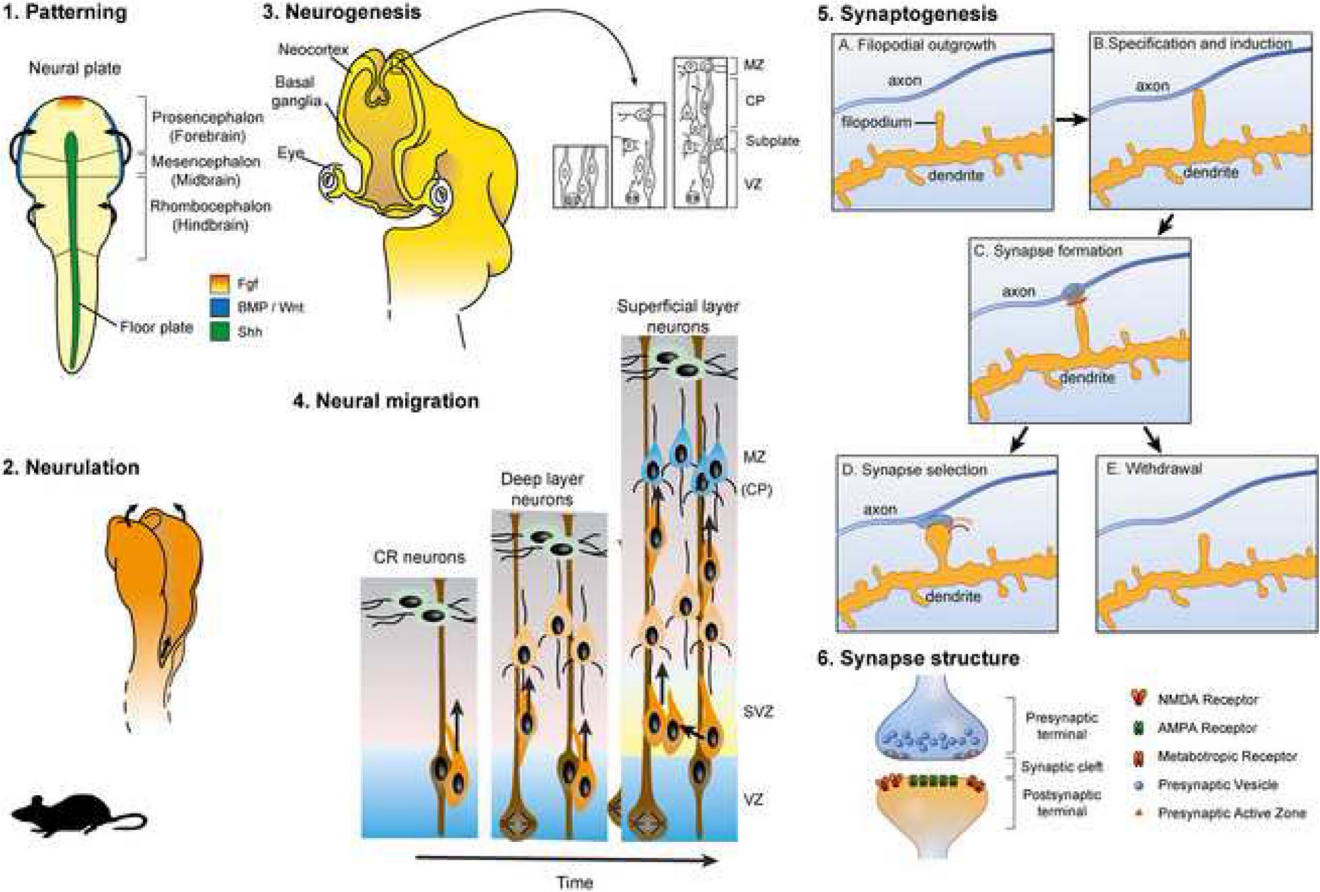
Embryonic development of the cerebral cortex: a primer. The development of the cerebral cortex can be divided into several stages (see figure). 1. *Patterning*: The basic plan of the mammalian brain is laid out at the neural plate stage. At this stage, signaling centers that surround the neural plate produce signaling molecules including FGFs, BMPs, and SHH which form a set of intersecting gradients across the neuroectoderm. Combinations of these signals are believed to confer specific regional fate on neural plate cells [61]. Next, in the process of *neurulation* (2), the edges of the neural plate fold towards each other and fuse together, thus forming the neural tube. At the earliest stages, the neural tube contains three primary brain vesicles, the prosencephalon (forebrain), mesencephalon (midbrain), and the rhombencephalon (hindbrain) [71]. The prosencephalon expands disproportionately, becoming larger than the other vesicles. Each vesicle subsequently gives rise to specific parts of the brain. For example, the prosencephalon will give rise to the cerebral cortex, ventral telencephalon, thalamus, and hypothalamus [152]. In the early neural tube, neuroepithelial progenitor cells divide symmetrically at the ventricular edge, giving rise to two daughter progenitors. These proliferative divisions rapidly expand the pool of neural progenitors [47, 122]. Neuroepithelial progenitor cells subsequently transform to form other progenitor types, primarily radial glia. Radial glia may divide either symmetrically or asymmetrically, giving rise to a radial glial and a neuronal daughter, thus initiating the process of *neurogenesis* (3) in which cortical neurons are born*.* As development proceeds, an increasing proportion of radial glia divide asymmetrically, generating large numbers of neurons. Another important population of neural progenitors, known as intermediate progenitors or apical progenitors, are found in the subventricular zone (SVZ) [47, 100, 101, 140]. Intermediate progenitors then continue to divide, making more neurons. Newborn neurons migrate (4) radially (indicated by black arrows) towards the outer (pial) edge of the embryonic cortex guided by a scaffold provided by radial glial fibers that project from the ventricular edge to the pial surface. Early-born neurons populate the deepest layers of the cortex. Later born neurons migrate past them, progressively building up the characteristic six-layered of the cortex, in the process of *lamination* [122, 123, 132, 140]. Neurons in each cortical layer have distinct molecular signatures, associated with their specific functions. Excitatory (glutamatergic) cortical neurons are generated in dorsal telencephalon, but inhibitory (GABAergic) cortical neurons are born in the ventral telencephalon, form where they migrate tangentially into the forming cortex. Once cortical neurons have migrated to their final destinations, they next form connections with their appropriate synaptic partners in the process of *synaptogenesis* (5), the first step in neural circuit assembly. During synaptogenesis, cell adhesion molecules such as neuroligins and neurexins are recruited to the site of the future synapse where they form a bridge between the axon and dendrite. This initiates protein specialization to organize the active zone of the presynaptic terminal and the post synaptic density (PSD) over a period of hours to days [106]. During this process, scaffolding proteins such as membrane-associated guanylate kinases (MAGUKs), PSD95, and SHANK1 are recruited to the site of axo-dendritic membrane contact [14, 69]. Next is the process of *synapse stabilization* (6). In rodents, thousands of synapses and dendritic spines per neuron are added in the period of 1-2 weeks of development but the majority of the synapses are removed or withdrawn and neuronal activity plays a key role in this [54, 109]. Many of the proteins located in the developing PSD play a role in synapse stabilization as many were shown to regulate synapse number and size. Neuronal connections could be between neurons from other brain structures that are further away (long-ranged connectivity) or with neurons from the same region of the brain (local connectivity). These connections are not final as many connections are made throughout embryonic and early development of the brain which will then be refined later on in development as connections that are used more are strengthened (activity-based neural connections) while connections that are less used are pruned as describe in the process of synapse stabilization to establish mature neural circuits [131, 142]. Figure is modified, with permission, from Price et al. (2017)

One such point of convergence is synaptogenesis and synapse physiology [8, 33, 56]. One of the earliest ASD-risk genes identified, *SHANK3*, and many other ASD-risk genes identified subsequently are directly involved in synapse physiology, highlighting the clear role that dysregulation of synaptogenesis and synaptic transmission play in ASD pathophysiology. So, ASD has often been viewed as a disorder of synaptic dysfunction [160]. However, many neurodevelopmental processes which occur during embryonic and fetal stages, such as neurogenesis and cortical lamination, are also a point of convergence for ASD risk genes, indicating that ASD can arise from an earlier point in development [16, 110, 145]. This view is supported by a recent genome-wide association study (GWAS) which showed that a large proportion of ASD risk genes analyzed were expressed most highly during fetal corticogenesis [55]. These early stages of brain development are highly dynamic. One could hypothesize that small changes during these processes could lead to larger effects later. Key stages of brain development at which ASD-related genes may act are described in Fig. 1.

Studying human prenatal brain development directly remains a major challenge, due to scarcity of material and ethical constraints on research using human embryos. Much of our present understanding of brain development is therefore based on studies using model organisms, primarily the mouse. However, there are important differences between mouse and human brain development [47]. The recent advent of cerebral organoids offers the potential to study human brain development directly. Cerebral organoids are 3D-cultured cell aggregates derived from pluripotent stem cells (PSCs) which closely resemble embryonic brain tissue. They contain many of the cell types found in embryonic brains, locally organized in a similar way to that found in vivo, and exhibit similar behaviors [78], but the spatial organization along major axes (anteroposterior, dorsoventral, and mediolateral) found in embryos is absent in organoids. Organoids have the potential to be invaluable tools for studying both normal development and the developmental origins of neurodevelopmental disorders including ASDs. A number of studies have already used organoids to model neurodevelopmental disorders as summarized in Table 1.

**Table 1:** Summary of neurodevelopmental disorders modeled using cerebral organoids. Several studies have identified cellular phenotypes of neurodevelopmental disorders using organoids. Here, we summarize the organoid model used, the phenotypes found, and the age at which they were detected

Disorder	Mutation	Organoid type	Time-point(s) analyzed	Cellular phenotype identified	Reference
Microcephaly	CDK5RAP2^+/−^	Cerebral	30 days	Imbalance of symmetrical/asymmetrical division	[78]
ASD	Idiopathic	Cerebral	44 days	FOXG1 overexpressed in ASD	[89]
ASD	CDH8^+/−^	Cerebral	50 days	Dysregulation of neurogenesis associated genes	[149]
Miller-Dieker syndrome	17p13.3 del	Cerebral	45 days	Reduced migration	[6]
Timothy syndrome	CACNA1C, GoF	Forebrain assembloid	80 days	Migration and depolarization defects	[9]
Angelman syndrome	UBE3A^−/−^	Cortical	120-150 days	Hyperexcitability and synchronous firing	[139]
Schizophrenia, autism	DISC1^+/−^	Cortical sliced	120 days	Lamination defects	[120]

*GoF* gain of function, *del* deletion

Protocols for generating human cerebral organoids fall into two main categories. In the first, PSCs are aggregated and allowed to differentiate in the absence of any specific added differentiation cues. Such protocols, exemplified by [78], produce heterogeneous organoids containing areas corresponding to various regions of the brain, such as dorsal and ventral forebrain, hindbrain, hippocampus, or choroid plexus. Alternatively, many protocols include the addition of specific cues that promote formation of a specific region of the brain, such as dorsal forebrain, ventral forebrain, midbrain, hypothalamus, or thalamus [9, 119, 154]. Such regionalized organoids can be used to investigate developmental patterning and the effects of mutations on individual brain regions. A recent study summarized all widely used organoid protocols and compared the transcriptomic profiles of the organoids grown using a range of published protocols. They found that while each protocol produced organoids with similar cellular composition, the differentiation trajectories differed between protocols [141]. Before using cerebral organoids to investigate normal development or disease states, it is important to understand how accurately they can recapitulate the in vivo system and what their limitations are. Comparing the transcriptomes of cortical organoids with those of the human fetal cortex shows encouraging similarities. At a global level, the transcriptome of dorsal forebrain organoids grown for 40-100 days correlated best with fetal cortex tissue at ages 8-16 p.c.w., indicating that organoids develop at a similar rate to the fetus [1, 86, 89]. The epigenome of organoids is also similar to that of fetal tissue—analysis of histone modifications showed cortical organoids were more like fetal brain tissue than adult brain tissue or pluripotent stem cells [1].

Single-cell analysis of telencephalic organoids and fetal human cortex showed that they contain very similar cell types. While excitatory neurons are the most numerous cell type within the cortex, several other vital cell types are also present. Fetal cortex at ages 15-23 p.c.w. contains, in decreasing order of proportion: excitatory neurons, inhibitory neurons that migrated from the ventral telencephalon, radial glial, astrocytes, microglial, with a small subset of glia, endothelial cells, oligodendrocyte progenitor cells, and Cajal-Retzius cells [1, 40, 159]. At early stages (1-3 months in culture), human dorsal forebrain organoids contain mostly excitatory neurons, radial glial, and intermediate progenitor cells [1, 9, 147, 155], while at 6 months there is an increase in astroglia and inhibitory interneurons (possibly of the olfactory bulbs) begin to appear [147]. Ventral patterned cortical organoids, on the other hand, contain GABAergic neurons, ventral progenitors, and astroglial cells, the key cell types found in the cognate brain structures in vivo. Although organoids show similar cell type composition to that found in vivo, a recent large scRNA-seq analysis of organoids and primary tissues, showed organoids lacked cell type and sub-type fidelity indicated by co-expression of different cell-type markers in organoids when compared to the fetal brain [7].

Organoid transcriptomes change during differentiation in accordance with the development of the fetal cortex. Modules of genes co-expressed during cortex development in vivo are conserved in organoids. These include upregulated genes associated with synaptic transmission, cell adhesion, neuron differentiation, and downregulated cell cycle genes [1, 86]. Notably, several gene modules co-expressed during organoid development are enriched with SFARI genes, a curated list of genes associated with ASD, and a quarter of SFARI genes are differentially expressed during organoid differentiation [1]. In vivo, genes linked to ASD and intellectual disability, including genes associated with chromatin remodeling, Wnt, and Notch signaling, were most highly expressed at 8-16 p.c.w [65]. Another single-cell transcriptome analysis found around 84% of genes mutated in disorders affecting neurogenesis showed the same developmental expression trajectory in organoid and fetal cells [15]. These studies suggest that organoids can recapitulate the timeline of events during normal human development during which ASD causative effects could be taking place.

## Possible developmental origins of ASD

### Abnormal neurogenesis and growth of the cerebral cortex

**Fig. 2:**
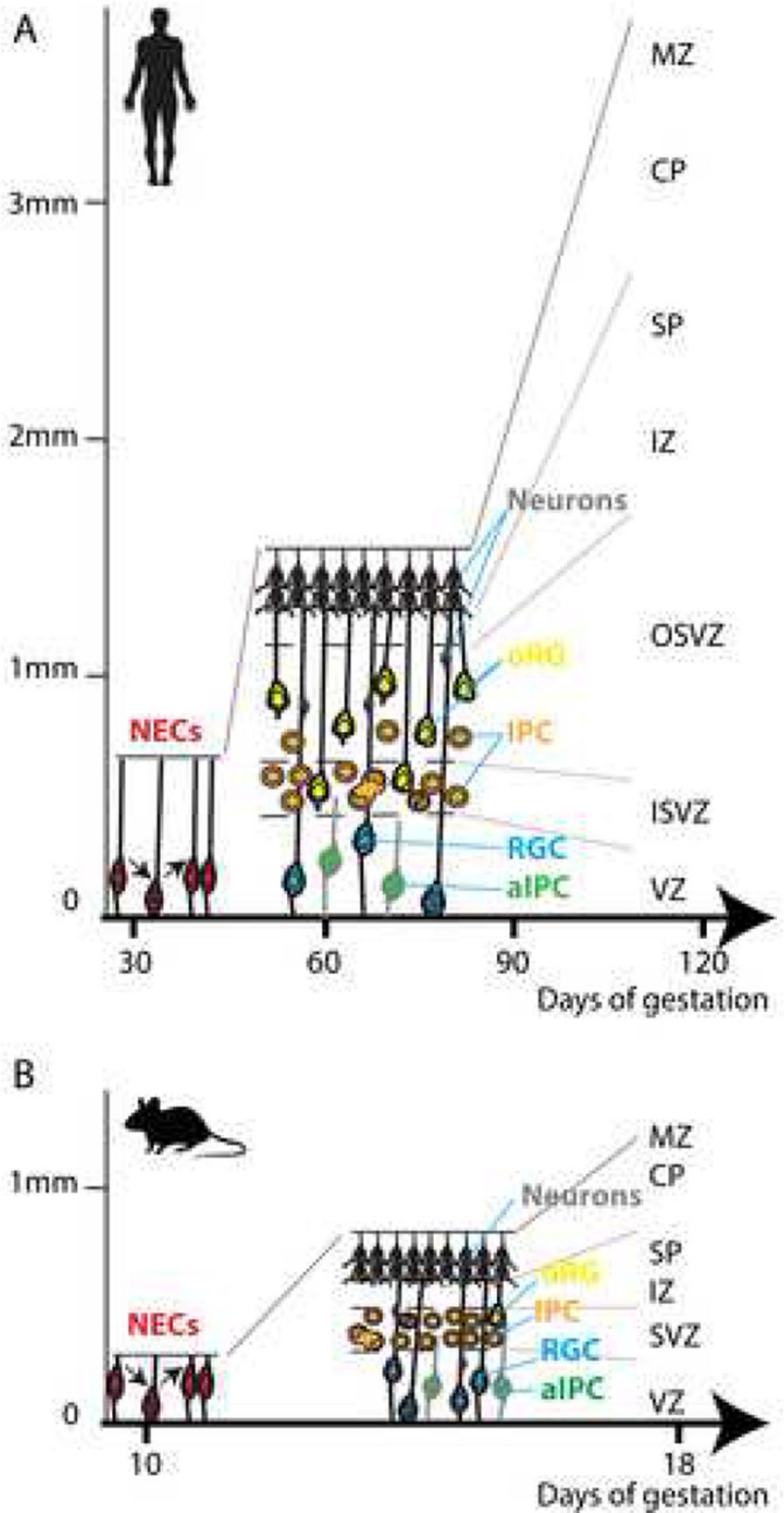
Cortical expansion in humans. There are significant differences in the number, types, and behavior of cortical neural progenitors between mouse and human (see figure). In mouse, early neuroepithelial cells (NEC, colored red) divide symmetrically, to increase the size of the neuroepithelium before transforming to become radial glial progenitors (RGC, colored blue). RGCs and apical intermediate progenitor cells (aIPCs, colored green) initially self-renew but gradually begin to undergo asymmetric divisions to produce either an intermediate progenitor cell (IPC, colored orange) or a neuron (grey) [41]. Most IPCs divide only once, producing two neurons. Once the balance of radial glial divisions shifts from self-renewal toward differentiative divisions, final neuronal output becomes restricted. Newborn neurons migrate radially through the intermediate zone (IZ) and subplate (SP) before settling in the cortical plate (CP). Equivalents of each of these progenitor types are found in primates, where they show increased self-renewal which, along with an increased starting population, leads to a larger VZ and SVZ [136, 156]. Primates, including human, have two proliferative subventricular layers, the inner and outer subventricular zones (iSVZ and oSVZ respectively). The oSVZ contains a variety of highly proliferative progenitor cells including outer radial glia (oRG, colored yellow) which give rise to large numbers of cortical neurons [45, 58]. oRG are abundant in human embryonic cortex, but extremely rare in mouse. Further, neurogenesis takes place for longer in human cortex compared to mouse, allowing more rounds of cell division. Overall, the combination of higher starting cell population, additional progenitor types, higher proliferative capacity of progenitors, and longer time-window for neurogenesis have contributed to the large expansion of human cortex compared to mouse. Figure is modified, with permission, from Mason and Price (2016)

One obvious difference between mouse and human brains is the disproportionately increased size of the human cerebral cortex. The mechanisms that led to the dramatic expansion of the human cortex are outlined in Fig. 2. Enlarged head size is a common feature of ASDs. Some 14%–34% of autistic patients show macrocephaly [127], due to increased surface area rather than an increased cortical thickness [105]. Increased brain volume has been linked to the emergence and severity of autistic social deficits [59]. Analysis of head circumference of children with ASD over the first year of life showed an accelerated increase in head size [28]. Aberrant brain growth could be due to changes to the balance between proliferation and differentiation of neural progenitor cells in the embryo [29, 47]. Cell-cycle genes have been implicated in ASD based on differential expression in postmortem ASD brains [23]. A systems biology approach analyzing total brain volume and gene expression levels (in blood, given the unavailability of brain tissue) in ASD toddlers also implicated cell-cycle genes in regulation of brain size [117]. Mutations in cell cycle control genes have been found in ASD patients. For example, mutations in the transcriptional regulator *ANKRD11* (Ankyrin repeat domain 11) contribute to ASD [68, 90]. *ANKRD11* regulates neural progenitor proliferation through interaction with histone deacetylases [157]. Mutations in *PTEN* (phosphatase and tensin homolog on chromosome ten), a phosphatase that acts to inhibit the AKT/mTOR pathway, are associated with macrocephaly [13] and ASD [26, 35]. *Pten* heterozygous mutant mice have an increased number of neural progenitors and a decreased total cell number in the mature cerebral cortex, due to fewer neural progenitors exiting the cell cycle [22]. In contrast, *PTEN* homozygous mutants in human cerebral organoids exhibited an expanded VZ and oSVZ, delayed neuronal differentiation, and surface expansion and folding [81].

Numerous mouse studies demonstrate that mutations in cell cycle genes can cause overgrowth of cerebral cortex. For example, knockdown of *Ankrd11* by *in utero* electroporation at E13/14 revealed decreased neural progenitors and underproduction of neurons, an effect that was rescued by administering a histone acetyltransferase inhibitor [49]. Overexpression of Cdk4/Cyclin D1 via in utero electroporation of mouse brains inhibited the switch from proliferation to differentiation, resulting in a cortex with a larger surface area, reminiscent of the increased surface area found in human ASD patients [79, 115].

#### Progenitor proliferation and neurogenesis in human cerebral organoids

Human cerebral organoids faithfully recapitulate embryonic cortical structures—they generate neuroepithelium and PAX6+ SOX2+ progenitors are found at its apical surface, closely resembling the embryonic ventricular zone. An adjacent layer of TBR2+ intermediate progenitor cells indicates the presence of an SVZ, and expression of neuronal markers TBR1 and MAP2 at the basal surface indicate cortical plate [78]. The presence of an outer SVZ (oSVZ) as indicated by expression of outer RG markers HOPX and PTPRZ1 has also been reported in some cerebral organoids [119, 120]. This is important, as oSVZ progenitors are thought to drive cortical expansion in humans and are absent in mice. Thus, human organoids are likely to be an invaluable tool to study this population of cells.

Human cortical organoids have been used to study disorders with a brain size abnormality. Organoids grown from iPSCs derived from a patient with severe microcephaly had reduced neural tissue, resembling the patient phenotype. Analysis of the early stages of organoid differentiation showed they had smaller neuroepithelia, fewer radial glial cells, and more neurons indicating an imbalance of the symmetric and asymmetric divisions of neural progenitor cells [78].

In a study of idiopathic ASD, iPSCs were derived from family members with an ASD and unaffected close relatives, then cerebral organoids were grown from each. The transcription factor *FOXG1* was found to be significantly overexpressed in the ASD patient-derived organoids, driving an accelerated cell cycle [89]. Transcriptome analysis showed increased expression of genes associated with neural differentiation and synaptic transmission in ASD-organoids [89], both of which have been linked to ASD [35, 53, 116]. ASD-organoids showed increased neural maturation and surplus GABAergic neurons but no effect on excitatory neuron number, indicating an imbalance of excitatory/inhibitory neurons, a phenotype believed to underlie some cases of autism. Inhibition of *FOXG1* expression restored GABAergic neuronal numbers to normal [89].

*CHD8*, one of the most commonly mutated genes in ASD, can negatively regulate WNT signaling, an essential signaling pathway in brain development. Transcriptome analysis of forebrain organoids derived from *CHD8*^*+/−*^ iPSC lines and their isogenic controls showed dysregulation of genes associated with neurogenesis, Wnt signaling and ECM components [149]. Notably, there was a significant overlap of these differentially expressed genes when compared to those found in NPCs and neurons derived from *CHD8*^*+/−*^ iPSCs compared to controls in 2D culture experiments [148]. Similarly, 23% of differentially expressed genes found in *CHD8*^*+/−*^ organoids were also found in idiopathic ASD organoids, with these overlapping genes being enriched with neurogenesis-associated genes [89, 149].

### Defective neuronal migration/cortical lamination

Disruption to cortical lamination may be a common feature of brain development in ASD [16, 138]. Cortical layers form progressively during embryonic development, with deep layer neurons being born first and later-born neurons migrating past them to form the characteristic six-layered laminar architecture of the cortex. Defects in migration could be indirect effects of altered cell cycle dynamics or proliferation as migration defects are also observed in the mouse models of the genes discussed above. For example, in *Ankrd11* mutant mice, more cells are retained in the VZ and SVZ, resulting in fewer cells in the cortical plate. Furthermore, there were fewer Satb2-expressing superficial layer neurons and Tbr1+ deep-layer neurons were positioned inappropriately [135]. *Pten* heterozygous mutant mice showed an increase in superficial layer Cux1-expressing neurons [22].

The transcription factor *TBR1* is required for normal cortical lamination and has been implicated in ASD [5, 35, 64]. Tbr1 is expressed in deep-layer neurons (layer 5/6) and in *Tbr1*^*−/−*^ mutant mice, neurons in layer 5 and layer 6 of the cortical plate are mixed and there is no clear distinction between them [5, 64]. Tbr1 is required to maintain layer 6 identity in the postnatal cortex—specific deletion of *Tbr1* in layer 6 led to increased expression of regulators of layer 5 identity such as *Fezf2* and *Bcl11b* and a decrease in layer 6 markers/regulators *Foxp2* and *Tle4* [42].

Before initiating radial migration, newborn cortical neurons undergo a multipolar to bipolar morphology change. This transition is disrupted in mice deficient for a number of genes implicated in ASD including *Foxg1* [94] and *Fmr1* [75]. *Foxg1*, a transcription factor, is expressed in neural progenitors and has multiple roles in forebrain development in mice [87, 134]. Downregulation of Foxg1 expression is required to allow cells to progress from multipolar to bipolar morphology before migrating into the cortical plate [94]. Delay in multipolar to bipolar transition impairs the coordinated integration of excitatory neurons with inhibitory interneurons, ultimately affecting the ratio of excitatory to inhibitory neurons in the developing cortex [89, 94].

#### Neuronal migration and cortical lamination in cerebral organoids

Forebrain organoids contain radially aligned RGC processes as seen in vivo [118]. Live imaging of neurons migrating from organoids onto a Matrigel surface showed similar migration rates to ferret cortical explants [6]. Using this model, migration defects were observed in organoids derived from iPSCs from Miller-Dieker syndrome (MDS) patients; a severe cortical malformation disorder caused by defective cortical neuronal migration. Imaging analysis showed fewer neurons migrating out of the organoid, reduced migration speed, and track straightness [6].

Migratory defects can also be examined by using an “assembloid” approach. By co-culturing ventral telencephalic organoids with dorsal cortical organoids, interneurons from the ventral organoids were shown to migrate towards the dorsal forebrain as observed in vivo [9]. Using this model, assembloids were generated using hiPSCs derived from patients with Timothy syndrome (TS), a neurodevelopmental disorder characterized by ASD and epilepsy [9]. These assembloids exhibited migration defects in interneurons and increased residual calcium following depolarization in TS neurons. Timothy syndrome is caused by a gain-of-function mutation in an L-type calcium channel (LTCC) subunit and incubating organoids with an LTCC blocker successfully reversed the migratory defects [9].

**Fig. 3:**
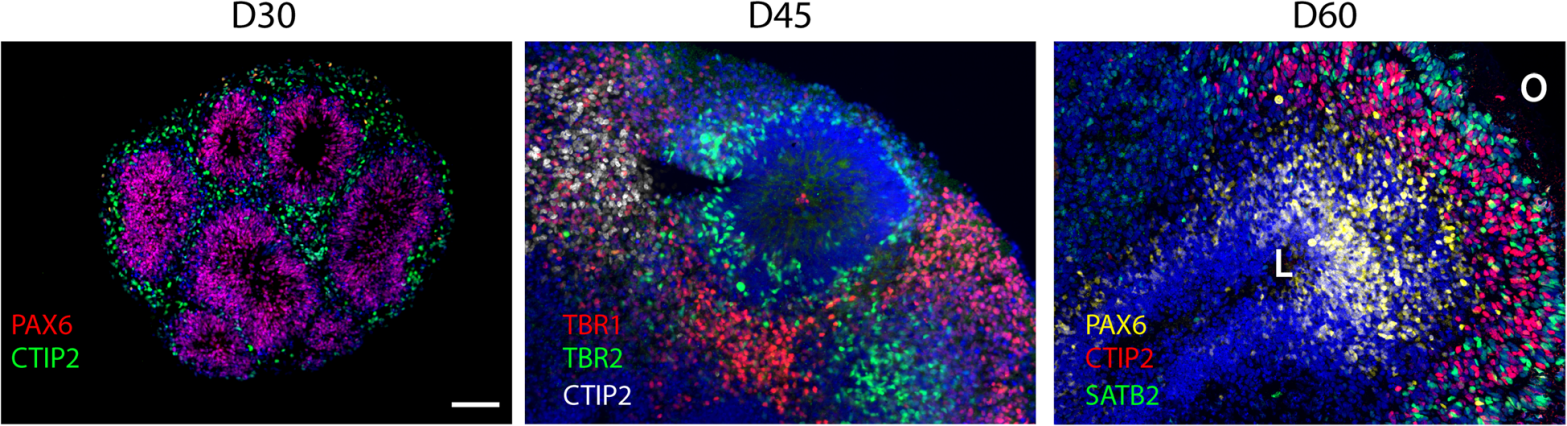
Sections of (**a**) 30-day, (**b**) 45-day, and (**c**) 65-day-old cerebral organoid grown from wild-type human iPSCs. **a** Immunostaining for progenitor marker PAX6 (red) and deep layer neuronal marker CTIP2 (green). **b** Immunostaining for intermediate progenitor marker TBR2 (green), deep later neuronal marker TBR1 (red), and deep layer neuronal marker CTIP2 (white). **c** Immunostaining for progenitor marker PAX6 (yellow), deep layer neuronal marker CTIP2 (red), and upper layer neuronal marker SATB2 (green). Progenitors are located interiorly, near the central lumen whereas differentiated neurons are located towards the outer edge of the organoid and multiple time points show that neuronal layers are progressively established, resembling in vivo embryonic cortex. L, lumen, O, outer edge. Scale bar, 100 μm

Human cortical organoids show some lamination (Fig. 3), with progenitor cells located towards the central lumen and differentiated neurons located towards the outside. Expression of TBR1, CTIP2, and SATB2 showing similar lamination in day 84 organoids as found in neocortex at 23 GWs [119, 128]. A recent study showed that cutting organoids into thick organotypic slices, thereby improving nutrient access from the culture medium, greatly enhanced lamination as indicated by larger and more distinct oSVZ and CP layers. Using this method, they identified lamination defects in organoids with a *DISC1* mutation, which has been associated with schizophrenia and autism [73, 120].

### Abnormal synaptogenesis in ASD

Following neurogenesis, neuronal migration and cortical lamination, neurons next make connections with their appropriate synaptic partners, thus beginning the formation of neural circuits. This is known to be important in the pathophysiology of ASD as synaptogenesis is another point of convergence for ASD-risk genes [27, 33]. Here, we discuss some of the well-known ASD-risk genes involved in synaptogenesis, but this is by no means an exhaustive list. A more comprehensive list is provided in a recent review by Guang and colleagues [56].

Some of the first genes implicated in ASD, such as the multiple Ankyrin repeat domain 3 gene *SHANK3*, are directly involved in synapse formation and function. *SHANK3* was found to be disrupted in a child affected with Phelan-McDermid syndrome (PMS) in which patients show poor eye contact, global developmental delay, decreased socialization, and stereotypic movements [10]. Analysis of more than 60 additional patients showed that heterozygous loss of *SHANK3* is responsible for the neurological phenotypes [2, 36]. Subsequently, *SHANK3* mutations have been found in numerous ASD patients [37, 50, 95]. The SHANK protein family comprises of SHANK1, 2, and 3, scaffold proteins which localize to synapses and interact with components of the postsynaptic density including guanylate kinase-associated protein (PSD95), Homer, cortactin-binding protein, and the somatostatin receptor and act to stabilize the PSD-95/Shank/Homer complexes at the postsynaptic density [83].

In mice, Shank1 is required for correct synapse maturation and function. Deleting Shank1 leads to smaller dendritic spines, thin postsynaptic densities, and ultimately weakened synaptic transmission. Mutant mice showed impaired contextual fear memory, poor long-term retention of a spatial task, and anxiety-like behavior [67]. Shank1’s role in synapse maturation and function was corroborated in in vitro studies where Shank1 was overexpressed in hippocampal neurons, leading to increased maturation and size of dendritic spines [129]. Mice heterozygous for *Shank3* showed reduced basal synaptic transmission in hippocampal CA1 neurons and decreased long-term potentiation, an important mechanism in retaining nascent synapses [11]. Deleting *Shank3B* caused reduction in postsynaptic proteins such as Homer and PSD93. The thickness and length of the postsynaptic densities were also reduced in addition to lowered spine density. This was accompanied by a reduction in excitatory synaptic transmission in the striatum. The mice displayed anxiety-like behaviors and decreased social interactions similar to human PMS patients [113]. In vitro studies knocking down *Shank3* in hippocampal neurons led to a lower number but increased length of dendritic spines supporting evidence from the mouse model that Shank3 is crucial for dendritic spine function [126]. Furthermore, expressing *Shank3* in aspiny cerebellar granule neurons was found to be sufficient to induce functional dendritic spines [126]. Recent studies using human iPSCs with *SHANK3* knockdown also found defects in excitatory and inhibitory synaptic transmission [66].

Fragile X syndrome (FXS) is an ASD caused by insufficient expression of the *FMR1* gene. Symptoms include intellectual disability, motor abnormalities, anxiety, speech delay, gaze avoidance, and stereotyped repetitive behaviors [57]. Postmortem neuropathological studies on FXS patients revealed structural defects of dendritic spines [25, 70]. *FMR1* encodes FMRP, a multi-functional mRNA binding protein involved in the transport and localization of a subset of dendritic mRNAs [3, 76, 82]. FMRP is enriched in neurons and especially at the dendrites, where it represses the translation of many mRNAs that play important roles in synapse formation and synaptic plasticity [12, 31, 43, 93, 150]. FMRP represses the translation of many mRNAs [104] leading to an increase in rate of basal protein synthesis in the hippocampus of Fmr1 null mutant mice [108, 121].

Rett syndrome is an ASD in which patients appear to develop normally up to 6—18 months of age but then head growth decelerates, leading to microcephaly by the second or third year of life. Other symptoms include loss of language, social withdrawal, lack of eye contact, lack of response to social cues, and stereotypic hand movements [19]. Most cases of Rett syndrome are caused by mutations in the MECP2 gene, which encodes Methyl-CpG-binding protein 2, a nuclear protein that binds to methylated 5-hydroxymethylcytosine or CpG sites required for chromatin organization and transcriptional regulation [63, 80, 91]. MECP2 expression is most abundant in neurons with the expression level increasing postnatally as neurons mature [133]. MECP2 regulates expression of brain-derived neurotrophic factor (BDNF) which is a critical synaptic maturation factor maturation [18, 72, 103, 144]. MECP2 is important for the development and the maintenance of synapses. It is essential for the transcription of biosynthetic enzymes crucial for neurotransmitters in respective neurons such as tyrosine hydroxylase in catecholamine neurons, GAD in inhibitory neurons, and neuropeptides important for neuronal physiology such as corticotropin-releasing hormone, BDNF, and somatostatin [20, 130]. Rodent models lacking functional MeCP2 reproduce features of Rett syndrome patients [21, 112, 114, 153]. Mouse models also showed a decrease in synaptic density and reduced LTP and synaptic plasticity [96]. Similarly, in vitro data from neurons derived from human iPSC of Rett syndrome patients revealed decreased spontaneous postsynaptic currents with fewer synapses [88].

The finding that MECP2 overexpression or underexpression leads to Rett syndrome-like phenotypes further complicates the role of MECP2 suggesting that gene dosage is an important factor in ASD. This also suggests that ASD might reflect a failure of homeostatic regulation of synaptic function which makes sense as an optimal synaptic function only occurs within a narrow dynamic range. Too much or too little of a protein might tilt the balance out of this range, resulting in ASD.

## Organoids generated by current protocols are not mature enough for a thorough investigation of their electrical properties

Neuronal activity has been found in human organoids older than 3 months. Immunocytochemistry shows co-localization of pre- and post-synaptic markers in cortical organoids suggesting the formation of synapses [9, 111, 147, 154]. Action potentials have been recorded in 50-80% of neurons within organoids in response to depolarization [9, 111, 154] and spontaneous firing has also been observed in organoids, which is lost after incubation with neurotransmitter antagonists [9, 111, 143, 154]. Furthermore, deep layer neurons in fetal neocortex show complex dendrite morphology at GW26 [159], and neurons derived from stem cells display similar morphology 9 months after transplantation into the mouse cortex [38].

However, despite the presence of synapses and action potentials, there is as yet no robust evidence for functioning neural networks with anatomically correct circuitry. This could be because organoids lack the dorsoventral, anteroposterior, and other axes found in embryonic brains, so although normal neuronal types differentiate efficiently in organoids, they are unlikely to be arranged in the appropriate anatomical locations relative to their prospective synaptic partners. Further, axonogenesis and synaptogenesis do not become prominent in human embryos until around 5-7 months [32, 159], suggesting organoids are too immature. Some studies have characterized the electrophysiological properties of neurons in the human fetal cortex. One study using whole-cell patch clamping on slice culture of the prefrontal cortex found no action potentials detected in neurons at GW23 and only detected APs at GW26 [159], coinciding with the expression of axonogenesis genes during GW19-26.

While most organoid studies use transcriptomics to characterize their organoids, the presence of a transcript does not always indicate the presence of functional protein. Expression of NMDA receptor subunit mRNA was detected in young human fetal neurons but its protein was not detected until > 23 weeks [39]. Therefore, the expression of proteins in organoid neurons is likely a better marker of neuronal maturity.

Some electrophysiological properties, such as increased capacitance and sodium and potassium currents, mature over time in organoids, indicating neuronal maturation with age [119]. Analysis of 10-month-old organoids using multi-electrode arrays (MEA) showed consistently increased firing rates, burst frequency, and synchrony indicating maturing neural networks. Notably, comparison of timing of electrical activity of pre-term human EEGs with the MEA recordings from cortical organoids showed organoids over 6 months old had high developmental age correlation, suggesting that the organoids follow an intrinsic developmental timeline [143]. In a separate study, higher firing frequency was observed in thalamic organoids when they were fused to cortical organoids, suggesting that interactions between the organoids led to neuronal maturation [154]. Generating regionalized organoids may be limiting their maturation as extrinsic signals such as guidance molecules from neighboring regions may be required for full maturation.

Human ESC-derived neurons transplanted into mouse brain integrated and matured over a period of several months, eventually generating action potentials similar to those seen in adult humans. Intrinsic properties improved over time; membrane potential hyperpolarization, increase in maximum sodium currents, increased firing rates, dendritic length, and dendritic spine density. Two-photon and calcium imaging experiments found an increase in calcium activity in human neurons during mouse visual stimulation indicating synaptic integration within the host cortex [85].

These experiments show that stem cell-derived neurons can mature to produce functionally similar neurons to those seen in vivo [85, 143] and can functionally integrate with local circuitry [85]. However, the process of maturation takes several months (6-10 months) [85, 99, 143] following their own intrinsic developmental timeline independent of in vitro differentiation protocol or culture conditions [85]. Extrinsic signals can improve maturation [154] indicating some culture conditions may be limiting cortical organoid maturation.

Despite their lack of maturation, the electrophysiological properties of cerebral organoids have been shown to model some aspects of disease phenotypes. For example, organoids harboring mutations implicated in Alzheimer’s disease (AD) displayed increased levels of neurotransmitter transporter proteins and increased AP firing rates. This hyperexcitability has also been seen in AD mouse models and human brains [51]. Hyperexcitability was also seen in organoids modeling Angelman syndrome (AS), a neurodevelopmental disorder partly characterized by seizures. When compared to wild type, AS-cortical organoids showed increased firing with neurons in some organoids showing synchronous firing [139]. Changes in electrophysiological properties were also found in Timothy syndrome cortical organoids which displayed abnormal calcium signaling [9] and idiopathic ASD-cerebral organoids which required a more hyperpolarized membrane potential to inactivate sodium channels [89].

Organoids could well provide a useful tool for studying synaptic function in ASD but this will require improvements in differentiation protocols to allow normal maturation of neurons. This suggests that cerebral organoids, in their current form, are most appropriate for the study of earlier neurodevelopmental processes such as neurogenesis and cortical lamination rather than later processes such as synaptogenesis and neural circuit formation.

## Challenges in using cerebral organoids to model ASDs

### Differences between organoids and in vivo development

While there are many similarities between organoids and their in vivo counterparts, understanding the key differences can lead to improvements in the model. One major source of differences arises from the use of SMAD inhibition to direct stem cells toward neural fate. The human brain consists of more than just neural cells; non-ectodermal cells such as microglia, endothelial, blood cells, and immune cells make up around 23-27% of fetal cortex at ages 15-37p.c.w [40, 102].. Microglia make up ~ 20% of fetal cortex cells from scRNA-seq studies [40, 102], and may be involved in ASD pathogenesis by dysfunctional synaptic pruning [74]. Neuroimmunology is a large and expanding field as such many labs are working on ways to incorporate an immune system within the organoid model [107] reported that omitting SMAD inhibition gave rise to organoids containing all three germ layers, ultimately allowing microglia to develop within the organoid. Microglia can also become incorporated within organoids by co-culturing them with iPSC-derived microglia [84].

A major difference from the in vivo situation is the lack of vascularization in organoids, commonly leading to a necrotic core at their center. Most cells within day 44 cortical organoids were associated with glycolysis compared to just 2% in the fetal cortex, suggesting that organoid cells are metabolically stressed [1]. This abnormal rate of glycolysis could mask effects caused by ASD-causing mutations as studies have shown elevated glycolysis as a candidate cause of ASD [146]. Stress pathways were found to be upregulated in cortical organoids, irrespective of protocol, cell line, or organoid age [7]. Furthermore, these high-stress levels limit cell type specification of organoid cells [7]. Transcriptomic comparison between fetal tissues and organoids suggests that major differences are due to tissue culture environment and not due to differences in differentiation [15]. To improve nutrient and oxygen diffusion, spinning bioreactors [78], shaking culture systems [77], and sliced organoids [120] have been used. Using an air-liquid interface culture improves organoid survival, morphology, and axon outgrowth [52]. Lack of vascularisation could also be the cause of the most striking difference between organoids and their in vivo counterpart, their size, with organoids being considerably smaller, possibly suggesting that processes involved in cortical expansion (Fig. 2) are not well recapitulated in organoids. Recently, Qian et al. [120] showed that sustained growth of organoids can be maintained by slicing organoids open to improve diffusion. Progenitor zones of sliced organoids continued to expand over time and were packed with NPCs, IPCs, and oRGs, which are key cell types involved in cortical expansion. Despite their higher proliferative capacity, organoids did not undergo gyrification, although this could be induced by embedding organoids in ECM [120].

### Sources of variation

One of the main challenges in using organoids is their variability. Different laboratories tend to have their own in-house protocol in the generation of organoids and protocols vary in their differentiation efficiency depending on which cell line is used [6, 9, 78, 154]. There are multiple sources of technical variation between organoid differentiation protocols, for example, recombinant proteins used to promote differentiation, Matrigel, and serum all have some batch effects during their production. Ideally, it would be helpful to have a systematic analysis of all published protocols and an agreed standardized protocol to be used in organoid generation. Nonetheless, significant efforts have been made to tackle this issue. A recent study comparing individual organoids generated from different lines and different batches using single-cell RNA-seq analysis showed very low organoid-to-organoid variability when careful quality control checks such as checks for neural differentiation were used during the process of organoid differentiation [147].

A common issue when using PSCs in cell culture, particularly for prolonged culture or during stressful events such as gene editing, is their propensity to acquire genetic changes which may alter their growth, transcriptome, and/or differentiation, ultimately confounding experimental results [92]. PSCs appear to incur non-random genetic abnormalities, the most abundant being chromosomal duplications or deletions. These can be assayed by karyotyping or qPCR [4]. More subtle mutations such as SNP or CNVs can also occur in PSCs with apparently normal karyotype thus SNP arrays and/or genome sequencing should be performed on all lines used during experiments [92]. However, these techniques may miss some genetic alterations thus it is important to use multiple clonal lines for experiments.

## Future perspectives

Organoids provide a powerful, amenable in vitro system to study normal embryonic human brain development and how it is disrupted in neurodevelopmental disorders. Advances in imaging technologies and gene editing have opened multiple avenues to dissect the roles of specific genes during human brain development using organoids. Using advanced imaging of cortical organoids, such as light-sheet live-cell imaging microscopy [62], will allow scientists to track in real-time the high dynamic processes of human neurogenesis.

However, there are still many challenges facing the use of cortical organoids for studying ASD. One of the most obvious is the lack of any behavioral readout from organoids. Currently, ASD is diagnosed solely on behavioral characteristics but organoid studies might elucidate cellular phenotypes/readouts that were missed from studies using other model systems, given that human neurodevelopment is still relatively understudied and there are important interspecies differences [124]. Recent advances in sequencing technology and data analysis have increased our ability to understand complex neurodevelopmental disorders such as ASD by finding converging pathways [116, 151]. Coupling that with organoid technology, one can imagine sequencing and bioinformatic analyses of ASD patient-derived cerebral organoids might provide new biomarkers for diagnosing ASD, instead of relying on behavioral phenotypes.

Synaptogenesis and neural circuit formation are significant points of convergence in ASD-risk genes. Electrophysiological studies of cerebral organoids are limited in their current state due to the long maturation time needed for circuit formation. This is not helped by culture conditions which appear to induce stress on the organoids, limiting, or even regressing neuronal maturity [7]. Despite these limitations, some cellular phenotypes of neurodevelopmental disorders have been modeled using cerebral organoids (Table 1). Moreover, now that we have identified stress factors that limit neuronal progenitor maturation in organoids [7], we can proceed to improve organoid culture conditions, for example by redesigning cell culture media to better support neuronal maturation or the use of microfluidics to enhance nutrient intake.

It is challenging to link genotype with behavioral phenotype in ASD as ASD-linked mutations often show incomplete penetrance. For example, only 26% of individuals carrying the 16p11.2 duplication are diagnosed with ASD, and around 37% of carriers of the duplication have no psychiatric diagnosis [98]. Furthermore, monozygotic twins only show a 70% ASD concordance rate [48]. Stochastic events and/or environmental differences during early brain development may make one twin more susceptible to the effects of the mutation, leading to a more severe phenotype. While we might consider the current variability in organoids to be a confounding element in interpreting experimental results, this variability could hold the key in understanding the stochasticity and/or environmental differences that we find in monozygotic twins. More systematic studies will have to be undertaken to explore this, but the advent of organoids allows us for the first time to test this hypothesis.

Although cerebral organoids contain most of the cell types found in the brain, some important cell types are absent, such as microglia, which normally originate outside the brain [7, 141]. Therefore, it is still difficult to model cellular aspects of ASD pathologies such as neuroinflammation (which requires the presence of microglia) or white matter abnormalities. However, there are already efforts in increasing the complexity of the cerebral organoid culture system through the use of co-culture with other cell types such as microglia (can be used to model neuroinflammation) [137] and co-culturing with different brain regions to study axon tracts (to study white matter abnormalities) [9, 30, 154].

Despite their current limitations, cerebral organoids provide us with a valuable additional tool to investigate the etiology of autism. As better high-throughput techniques such as scRNA-seq and osmFISH [24] are developed, more information can be obtained from the limited human fetal and embryonic samples. This increased human data, coupled with cellular data from 2D and maturing human cerebral organoid and behavioral data from animal models, will together give us better tools to understand ASD.

## Data Availability

Not applicable

## Abbreviations

AD: Alzheimer’s disease
AP: Action potential
AS: Angelman syndrome
ASD: Autism spectrum disorder
CP: Cortical plate
FXS: Fragile X syndrome
GW: Gestational week
GWAS: Genome wide association study
IPC: Intermediate progenitor cell
iPSC: Induced pluripotent stem cell
ISVZ: Inner subventricular zone
IZ: Intermediate zone
LTCC: L-type calcium channel
MZ: Marginal zone
OPC: Oligodendrocyte progenitor cell
oRG: Outer radial glia
osmFISH: Ouroboros single-molecule fluorescence in situ hybridization
oSVZ: Outer subventricular zone
p.c.w.: Post-coital week
PMS: Phelan-McDermid syndrome
PSC: Pluripotent stem cell
RGC: Radial glial cell
scRNA seq: Single cell RNA sequencing
SFARI: Simons Foundation for Autism Research Initiative
SP: Subplate
SVZ: Subventricular zone
TS: Timothy syndrome
VZ: Ventricular zone
